# Using the Baidu index to understand Chinese interest in thyroid related diseases

**DOI:** 10.1038/s41598-022-21378-y

**Published:** 2022-10-13

**Authors:** Qian Hu, Yuan-lin Mou, Ruo-yun Yin, Lei Tang, Fan Zhang

**Affiliations:** School of Public Health, Research Center for Medicine and Social Development, Collaborative Innovation Center of Social Risks Governance in Health, Chongqing Medical University, No.61 Daxuecheng Middle Road, Shapingba District, Chongqing, 400016 China

**Keywords:** Diseases, Endocrinology

## Abstract

Common thyroid diseases are hyperthyroidism, hypothyroidism, thyroiditis, thyroid tumor and so on. Baidu is currently the most widely used online search tool in China, has developed an internet search trends collection and analysis tool called the Baidu Index. The aim of the present study was to understand the trend and characteristics of public’s online attention to thyroid diseases, and to explore the value of Baidu Index in monitoring online retrieval behavior of thyroid-related information. Taking the period from January 1, 2011 to December 31, 2019 as the time range into consideration, we used the big data analysis tool of Baidu Index and took “thyroid nodules”, “thyroid cancer”, “thyroiditis” “hyperthyroidism” and “hypothyroidism” as the keywords, the data of “search index” and “media index” were recorded on a weekly basis, and all information were aggregated into quarterly and annual to generate the final data which was carried out for secondary analysis. Pearson correlation analysis was used to analyze the correlation between the search index of keywords and the year. One-way Analysis of Variance was used to analyze the differences between search index and media index. Among the five keywords, thyroid nodule search index had the highest growth rate (640%), followed by thyroid cancer (298%). The media’s attention to thyroid diseases had been declining year by year. Unlike the public’s attention, the media index of hyperthyroidism was significantly higher than other keywords. Over the past nine years, the public's attention to thyroid-related diseases has been increasing gradually. Baidu Index is an effective tool to track the health information query behavior of Chinese internet users, which can provide a cost-effective supplement to traditional monitoring system.

## Introduction

Thyroid diseases (TDs) are a category of non-communicable disease that are easy to neglected, misdiagnosed and poorly managed^1^. At both ends of the spectrum, inadequate or excessive iodine intake can lead to thyroid disorders^2^. China was an iodine deficient country with a high prevalence of iodine deficiency disorders^3^, and in 1996, China implemented Universal Salt Iodization (USI) legislation nationally. During the 20 years of USI enactment, China has experienced excessive iodine intake (defined as median urine iodine concentration (UIC) ≥ 300 µg/L) for 5 years (1996–2001), more than adequate iodine intake (defined as median UIC from 200 to 299 µg/L) for 10 years (2002–2011), and adequate iodine intake (defined as median UIC from 100 to 199 µg/L) for 5 years (2012–2016)^4^. Since TDs are a health care and socio-economic burden in China, there has been increased interest in research on TDs intervention and prevention^5–7^.

The development of the internet has greatly changed people’s lives, especially the expansion of search engines, which has further enhanced the value of the internet as a tool for life, learning and work. According to the 49th Statistical Report on Internet Development in China, there were approximately 1032 million internet users in China by the end of December 2021, and the internet penetration rate reached 73.0%^8^. It was estimated that the utilization rate of search engine among netizens was about 81.3%. 77.3% of users could find the information they need through this service. Baidu search accounted for 90.9% of search engine users, ranking first^9^.

Baidu Index is a big data sharing platform constructed by massive user behavior information, which shows the search trend of the selected keywords, gains insight into the changes in the needs of netizens, monitors the trend of media public opinion, and locates the characteristics of users. The platform can provide data such as search index, demand map, information index, media index and population attributes. Currently, scholars have used Baidu index big data to analyze health data, and the research involves various aspects such as the assessment of online search trends and real demand for Lower urinary tract symptoms^10^, evaluation of outbreak monitoring prediction models for COVID-19 epidemics^11,12^, and prediction of the incidence of HIV/AIDS in China^13^. Through the analysis of these online search trend data, it is possible to reflect the pattern of health information search behavior and interest of internet users on population level. And there are no studies on TDs using the Baidu index yet.

This study used the Baidu Index data platform to obtain data and conduct secondary analysis in order to understand the characteristics of public attention to TDs, information search behavior and the trend of media attention, to explored the value of internet search data in monitoring online information search behavior. It provides a basis for meeting the public's need for understanding TDs, targeting the prevention and treatment of TDs, and complementing the advantages of traditional TDs surveillance systems.

## Methods

### Data from Baidu index

The data from Baidu Index (http://index.baidu.com/Helper/?tpl=helpandword=#pdesc) was used. Baidu Index is a big data sharing platform constructed by massive user behavior information, which shows the search trend of the selected keywords, gains insight into the changes in the needs of netizens, monitors the trend of media public opinion, and locates the characteristics of users. The platform can provide data such as search index, demand map, information index, media index and population attributes.

The data used in this study included: (1) search index: the data based on the search volume of netizens in Baidu, with keywords as statistical objects, scientifically analyze and calculate the weighted search frequency of each keyword in Baidu web search. (2) media index: the number of news reported by major internet media related to keywords and included by Baidu News Channel. (3) annual netizen search rate: search index/annual number of netizens (the annual number of netizens comes from the Statistical Report on Internet Development in that year).

The keyword “thyroid” was searched through the demand map of Baidu Index platform, and the weekly keyword demand map was collected in December 2019. The keywords related to TDs with the highest demand were selected: “thyroid nodule”, “thyroid cancer”, “thyroiditis”, “hyperthyroidism” and “hypothyroidism”. The two nouns of non-thyroid-related diseases: “what are the symptoms of thyroid” and “thyroid function” were excluded. The search index and media index for each keyword from January 1, 2011 to December 31, 2019 were obtained, a total of nine complete years. At the same time, due to the limitations of Baidu Index tools and the needs of research and analysis, this study recorded the data of search index and media index with weekly as the smallest unit, and summarized them to the quarter and year as the basis for subsequent data analysis.

### Statistical methods

In order to understand the characteristics and trends of public and media attention to TDs, we conducted an analysis by the following statistical methods. We added up the five keyword search indexes of each year to get the annual search index; the differences of annual search index, quarterly search index and annual media index of each keyword were analyzed by one-way ANOVA; the correlation between search index and year was analyzed by Pearson correlation analysis. After drawing the scatter plot and the regression line of the netizens' search rate in each year, the covariance analysis was conducted to test the statistical difference of the slope of the regression line among each group. *P* < 0.05 (two-tailed) was considered statistically significant. Microsoft Office Excel 365 (Microsoft, Redmond, WA, USA) and SPSS version 20.0 (SPSS, Inc., Chicago, IL, USA) were used to draw figures, and all statistics analyses were performed with SPSS.

## Results

### Changes in search index

**Figure 1:**
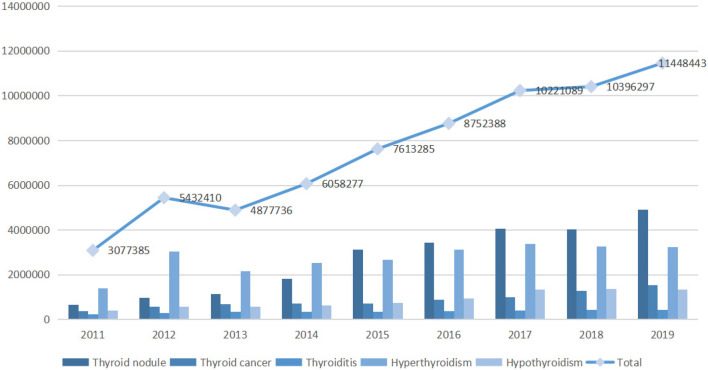
Changes in the annual search index.

Over the past nine years, the sum of the annual search index of each keyword showed an upward trend and was positively correlated with the year (Pearson's correlation = 0.983, *P* < 0.001).The Fig. 1 showed the changing trend of the annual search index. Each keyword was also positively correlated with the year (thyroid nodule: Pearson's correlation = 0.981, *P* < 0.001, thyroid cancer: Pearson's correlation = 0.956, *P* < 0.001, thyroiditis: Pearson's correlation = 0.934, *P* < 0.001, hyperthyroidism: Pearson's correlation = 0.784, *P* = 0.012; hypothyroidism: Pearson's correlation = 0.954, *P* < 0.001). In terms of search index growth, the absolute increase of thyroid nodule search index was the highest (4,236,537), followed by hyperthyroidism (1,845,562). The growth rate of thyroid nodule was the highest (640%), followed by thyroid cancer (298%). The changes of each search index over the nine years and their correlation with years were represented in Table 1.

**Table 1:** Basic situation and correlation analysis of search index from 2011 to 2019.

Keywords	2011	2012	2013	2014	2015	2016	2017	2018	2019	Increment	Increment rate (%)	Correlation coefficient	*P*
Thyroid nodule	662,208	964,825	1,140,236	1,808,814	3,122,765	3,425,706	4,071,499	4,034,230	4,898,745	4,236,537	640	0.981	< 0.001
Thyroid cancer	388,935	561,461	677,555	712,290	723,164	877,078	1,007,796	1,297,074	1,549,871	1,160,936	298	0.956	< 0.001
Thyroiditis	221,298	293,053	344,820	357,781	348,763	378,601	409,109	429,130	421,535	200,237	90	0.934	< 0.001
Hyperthyroidism	1,402,019	3,027,818	2,150,914	2,536,677	2,670,551	3,136,113	3,393,895	3,258,544	3,247,581	1,845,562	132	0.784	0.012
Hypothyroidism	402,925	585,253	564,211	642,715	748,042	934,890	1,338,790	1,377,319	1,330,711	927,786	230	0.954	< 0.001
Annual search index	3,077,385	5,432,410	4,877,736	6,058,277	7,613,285	8,752,388	10,221,089	10,396,297	11,448,443	8,371,058	272	0.983	< 0.001

Using the least-significant difference method, we found that there was a statistical difference between the search index of thyroid nodule and thyroid cancer, thyroiditis and hypothyroidism (*P* < 0.001), and between hyperthyroidism and thyroid cancer, thyroiditis and hypothyroidism (*P* < 0.001). However, there was no statistical difference between thyroid nodule and hyperthyroidism (*P* = 0.838). The search index of thyroid nodule surpassed that of hyperthyroidism for the first time in April 2015 and was higher than that of hyperthyroidism for four consecutive years; the search index of thyroid nodule and hyperthyroidism was always higher than that of the other three keywords in nine years. The specific results were shown in Table 2.

**Table 2:** Multiple comparisons between keywords (search index).

Keywords		*P*	95% CI	
Thyroid nodule	Thyroid cancer	< 0.001	1,058,300.69	2,571,433.53
	Thyroiditis	< 0.001	1,568,426.69	3,081,559.53
	Hyperthyroidism	0.838	−833,797.98	679,334.87
	Hypothyroidism	< 0.001	1,043,897.13	2,557,029.98
Thyroid cancer	Thyroiditis	0.181	−246,440.42	1,266,692.42
	Hyperthyroidism	< 0.001	−2,648,665.09	−1,135,532.24
	Hypothyroidism	0.969	−770,969.98	742,162.87
Thyroiditis	Hyperthyroidism	< 0.001	−3,158,791.09	−1,645,658.24
	Hypothyroidism	0.169	−1,281,095.98	232,036.87
Hyperthyroidism	Hypothyroidism	< 0.001	1,121,128.69	2,634,261.53

**Figure 2:**
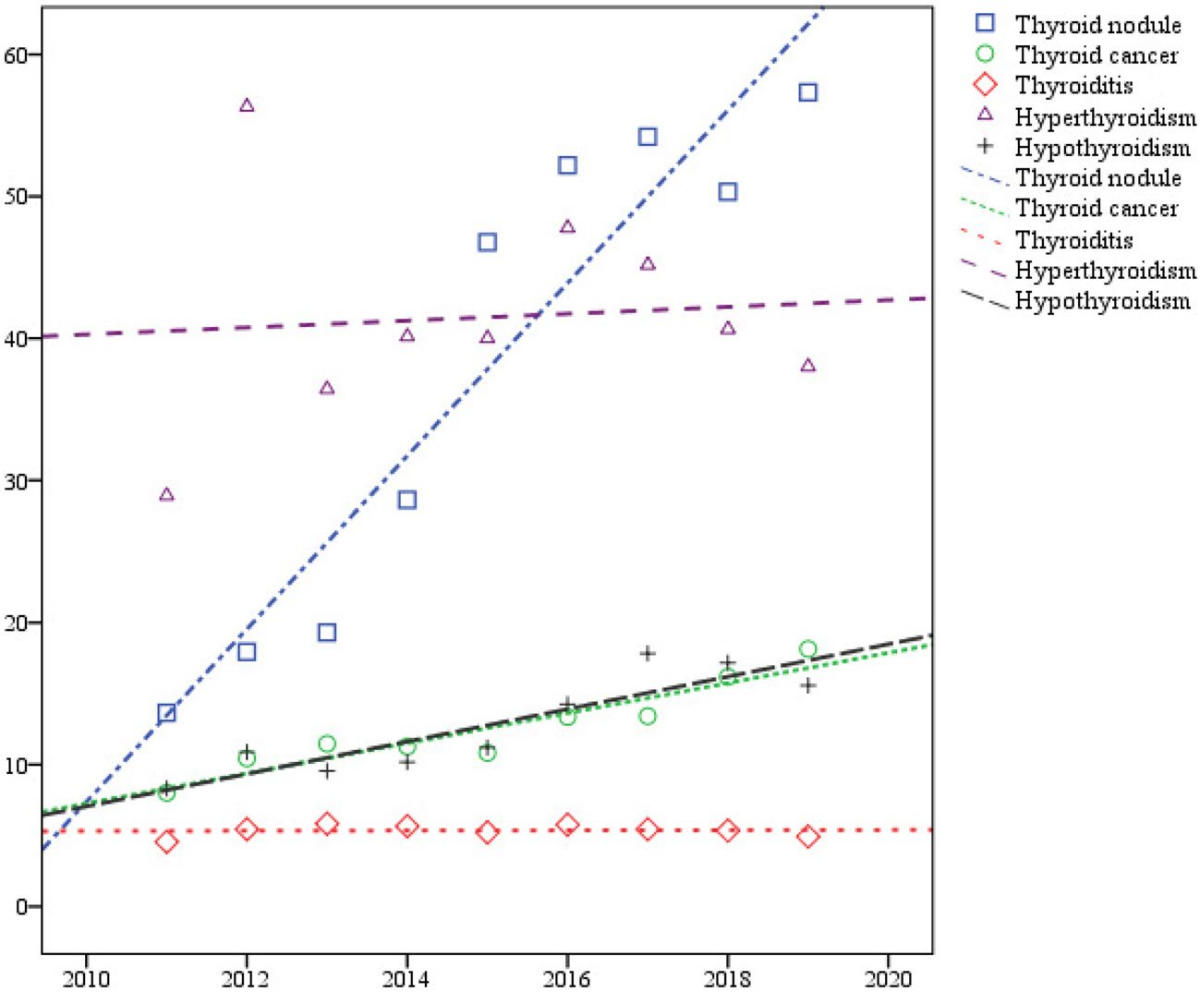
Scatter plot of annual netizen search rate.

As shown in Fig. 2, in the past nine years, the annual search rate of netizens showed an upward trend, and the regression linear slope of the five keywords was all greater than 0. The results of the covariance analysis showed that there was a statistical difference in the linear regression slope between different groups (F = 16.876, *P* < 0.001).

### Changes in media index

Unlike the keywords search index, the media index showed a downward trend in nine years (Pearson's correlation = −0.835, *P* = 0.005). The Table 3 showed the changes in the media index for each keyword over the nine years. Among them, the media index of hyperthyroidism was statistically different from that of thyroid nodule (*P* = 0.039), thyroiditis (*P* < 0.001), and hypothyroidism (*P* = 0.010). The relationship between other keywords were shown in Table 4.

**Table 3:** Basic situation of media index from 2011 to 2019.

Keywords	2011	2012	2013	2014	2015	2016	2017	2018	2019
Thyroid nodule	327	662	322	1336	638	201	170	134	250
Thyroid cancer	624	1382	371	481	651	445	204	199	218
Thyroiditis	173	282	129	280	13	0	0	0	0
Hyperthyroidism	1818	1237	1442	1960	672	380	126	120	190
Hypothyroidism	739	523	232	784	385	118	67	67	76
Media index	3681	4086	2496	4841	2359	1144	567	520	734

**Table 4:** Multiple comparisons between keywords (media index).

Keywords		*P*	95% CI	
Thyroid nodule	Thyroid cancer	0.772	−471.04	352.15
	Thyroiditis	0.092	−60.15	763.04
	Hyperthyroidism	0.039	−845.49	−22.29
	Hypothyroidism	0.570	−295.04	528.15
Thyroid cancer	Thyroiditis	0.050	−0.71	822.49
	Hyperthyroidism	0.073	−786.04	37.15
	Hypothyroidism	0.393	−235.60	587.60
Thyroiditis	Hyperthyroidism	< 0.001	−1196.93	−373.73
	Hypothyroidism	0.256	−646.49	176.71
Hyperthyroidism	Hypothyroidism	0.010	138.85	962.04

## Discussion

The results of this study showed that public attention to TDs had increased in the past nine years, but there were differences in different diseases. The attention of thyroid nodule and hyperthyroidism was significantly higher than that of hypothyroidism, thyroid cancer and thyroiditis, and the growth rate of thyroid nodule search index was more than twice that of the second place. Although all keywords showed an upward trend, the rising trend of thyroid nodule was more obvious than the other four keywords. This might be related to the increase in the prevalence of thyroid nodule in recent years^14^. The incidence of thyroid nodules was insidious, and most patients were asymptomatic in the early stage, and patients were more likely to inquire relevant information on their own after detecting discomfort^15^. As the largest search tool in China, Baidu's search results can reflect people’s needs well. The disease prediction product jointly developed by Baidu and the Chinese Center for Disease Control and Prevention can provide real-time data on infectious diseases^16^. At the same time, it can also be used to predict the epidemic trend of diseases, as a powerful complement to the traditional detection system^17^. Baidu Index has not been used for TDs related research in China. Our study is the first attempt to explore the behavior and interest of Chinese netizens in TDs, confirming the potential of using online search trend data to represent the real situation of TDs patients in China.

For the media index part, the results showed that the media's attention to the hyperthyroidism was higher than other keywords. This suggested that the media had pushed and reported more information about hyperthyroidism to the public in the past nine years. Overall, the media attention of TDs was on the decline. The reason might be related to the rapid development of the internet, the scattered news points, the shortage of media practitioners and the declined in the number of media concerned about TDs.

Despite the huge medical expenditure imposed on China by TDs^18^, due to China's vast territory and large population, it is difficult to evaluate the true prevalence rate of TDs and to understand the characteristics and needs of TDs patients. With the wide application of the internet and the increasing reliance of the public on search engines as the main way to query health information, some online digital diseases surveillance tools has been explored in recent years^19–21^. As a query tool, search engine can provide sensitive information on the disease before the diagnosis of the disease is reported, thus improving disease control. Internet big data has a broad application prospect in the medical field, which may be a supplement and an expansion of the current clinical and epidemiological data. Today, with the rapid development of the internet services and search engines, combined with network data analysis can be regarded as an auxiliary means of traditional disease monitoring.

### Limitations

This study also has several limitations. First, we only focused on the attention of Baidu search engine users to TDs, without considering the public attention on other search engines or social media, which can only reflect part of the public's attention to TDs. Second, there might be sampling biases in Baidu Index. Although the internet penetration rate in China had been greatly improved, the characteristics of internet users were obviously skewed to those with higher socioeconomic level and better educated segments. Meanwhile, although the target population of this study was Chinese, it was unavoidable that a small number of foreigners were included in the data. Third, although the data from the Baidu index were processed by a weighted filtering algorithm, the specific algorithm of Baidu Index has not been made public, so its validity and reliability cannot be assessed yet. Therefore, future research should consider including multiple search engines or social media for analysis to ensure the richness of the data. At the same time, the data should be mined in depth to control the influence of confounding factors on the study results and make the results more objective.

## Conclusion

Between 2011 and 2019, the online search rate of TDs maintained a sustained growth while the media index showed a downward trend. The Baidu Index can be used to track Chinese netizens' online behavior and interest in TDs. This may help to improve our understanding of the incidence of disease, patient education and the use of online resources. Internet search trend data is a valuable source for monitoring the search behavior of TDs-related information. It can be used as an exploratory tool to better understand the characteristics and preferences of patients and provide a scientific evidence for the control and prevention of TDs in China.

## Data Availability

The data analyzed in this study are availiable in Baidu Index Data Platform (http://index.baidu.com/Helper/?tpl=helpandword=#pdesc).

## Abbreviations

TDs: Thyroid diseases
USI: Universal salt iodization
